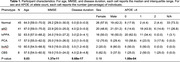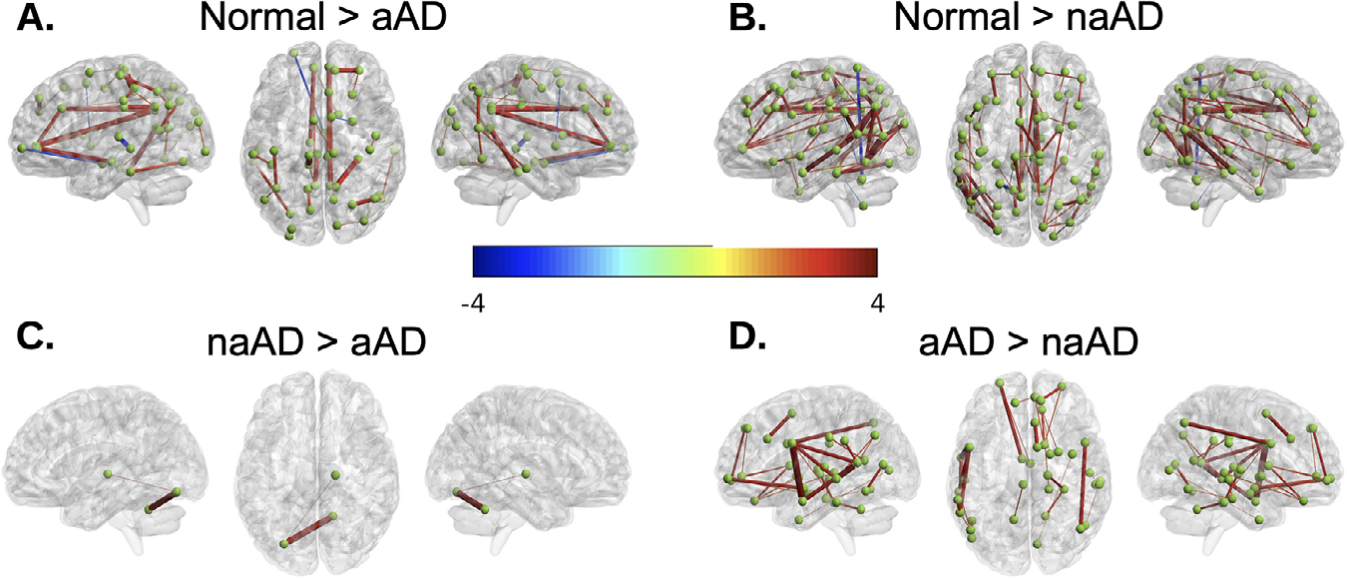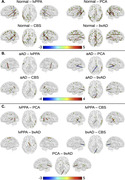# White matter integrity is lower in non‐amnestic than amnestic Alzheimer's disease and differs by clinical syndrome

**DOI:** 10.1002/alz.092815

**Published:** 2025-01-09

**Authors:** Jeffrey S Phillips, Nagesh Adluru, Moo K Chung, Hamsi Radhakrishnan, Christopher A Olm, Philip A. Cook, James C. Gee, Sanaz Arezoumandan, Katheryn A Q Cousins, David A Wolk, Corey T McMillan, David J Irwin

**Affiliations:** ^1^ Department of Neurology, University of Pennsylvania, Philadelphia, PA USA; ^2^ Penn FTD Center, University of Pennsylvania, Philadelphia, PA USA; ^3^ University of Wisconsin‐Madison, Madison, WI USA; ^4^ Waisman Laboratory for Brain Imaging and Behavior, University of Wisconsin‐Madison, Madison, WI USA; ^5^ University of Pennsylvania, Philadelphia, PA USA; ^6^ Penn Image Computing & Science Laboratory, Department of Radiology, University of Pennsylvania, Philadelphia, PA USA; ^7^ Penn Image Computing and Science Laboratory, Philadelphia, PA USA; ^8^ Digital Neuropathology Laboratory, University of Pennsylvania, Philadelphia, PA USA; ^9^ Penn Alzheimer’s Disease Research Center, University of Pennsylvania, Philadelphia, PA USA

## Abstract

**Background:**

Amnestic mild cognitive impairment and Alzheimer’s disease (aAD) exhibit degeneration of white matter (WM) tracts preceding overt cognitive decline. However, WM changes in non‐amnestic AD (naAD) are understudied. We hypothesized patterns of WM degeneration would differ between aAD and naAD.

**Methods:**

We compared WM degeneration, assessed by 30‐direction diffusion‐weighted imaging (DWI), in 41 individuals with aAD; 67 with naAD diagnoses including logopenic‐variant primary progressive aphasia (lvPPA), posterior cortical atrophy (PCA), behavioral variant AD (bvAD), and corticobasal syndrome (CBS); and 45 with normal cognition (Table 1). We performed deterministic tractography between 148 cortical/subcortical regions; connectivity was quantified by generalized fractional anisotropy (GFA). Tractwise GFA and regional grey matter (GM) volumes were converted to W‐scores adjusting for age and sex. We contrasted GFA between groups using regression models covarying for MMSE score. A mixed effects model was used to assess associations between tractwise GFA and volume of GM endpoints. Additionally, we computed the number of connected components in each person’s brain graph (i.e., number of subgraphs created by progressively stricter thresholds) across 100 GFA values. We contrasted this topological metric between groups and assessed its associations with MMSE and disease duration.

**Results:**

GM volume was lower in naAD and aAD than controls (both p<0.0001) but did not differ between patient groups (p=0.81). aAD and naAD patients had shared WM degeneration in corpus callosum, cingulum, and inferior/superior longitudinal fasciculi; however, WM degeneration was more severe in naAD (Figure 1). In lvPPA, WM degeneration was left‐lateralized; in PCA, GFA was reduced in bilateral parietal, occipital, and temporal areas (Figure 2). Across groups, GM and WM degeneration were significantly associated [F(1,20442)=326.0, p<0.0001]. Topological analysis indicated naAD but not aAD patients had lower global connectivity than controls. A higher component number (indicating reduced connectivity) was associated with longer disease duration and lower MMSE.

**Conclusion:**

Associations between GM volume and GFA suggest WM degeneration in naAD is related to AD pathologic change and not an independent process. Greater WM disease in naAD than aAD was not attributable to GM atrophy or symptom severity. Finally, topological metrics may serve as objective markers of global disease progression.